# Supplementary material for: Identification of Plasmodium GAPDH epitopes for generation of antibodies that inhibit malaria infection
Source: Life Sci Alliance. 2018 Sep 18;1(5):e201800111. doi: 10.26508/lsa.201800111 (PMC6238388; doi:10.26508/lsa.201800111)

- **Fig 4F-gel.** Wet stained gel scanned in B/W mode for better contrast.
- **Fig 4F-1.** Western blot using anti-thioredoxin Ab (expression tag). This is a replicate experiment (not shown in manuscript Fig. 4F) of Fig 4F-2.
- **Fig 4F-2.** Western blot using anti-thioredoxin Ab.
- **Fig 4-anti-CD68.** The membrane shown in Fig. 4F-2 was further incubated with anti-CD68 antibody and anti-mouse IgG secondary antibody. The anti-CD68 recognizes a 100~110 kDa protein (boxed). This was cropped to exclude the strong background bands of mouse IgG used for immuno-precipitation.

➢ *[Alkaline phosphatase]-conjugated secondary Abs were used for colorimetric detection for all Western blots . The boxed areas are shown in manuscript Fig. 4F.*

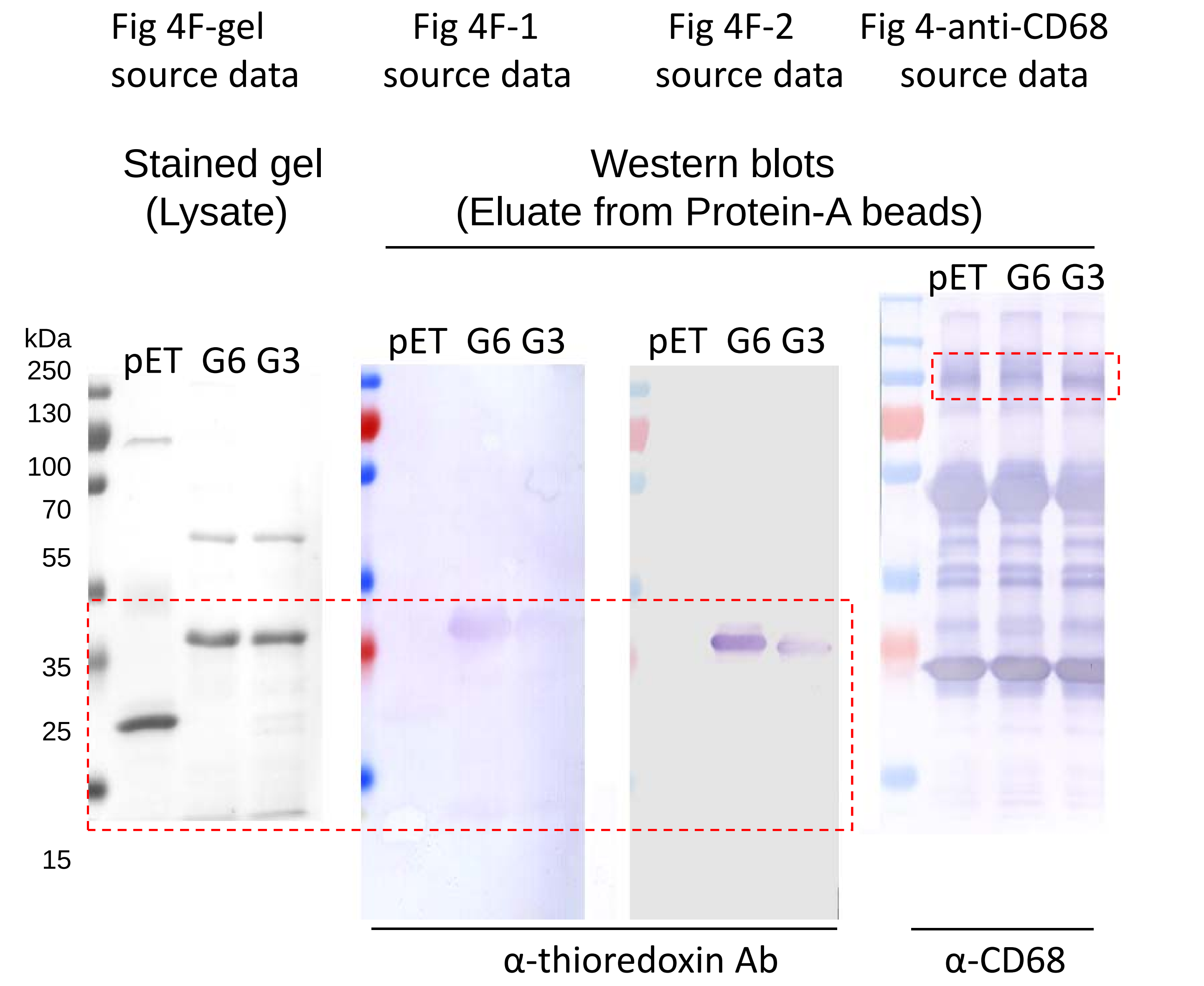

Supplement: Supplementary file 1 [file LSA-2018-00111_SdataF1.zip › LSA-2018-00111_SourceData_03.pdf]
