# Supplementary figures and images for: Identification of Plasmodium GAPDH epitopes for generation of antibodies that inhibit malaria infection
Source: Life Sci Alliance. 2018 Sep 18;1(5):e201800111. doi: 10.26508/lsa.201800111 (PMC6238388; doi:10.26508/lsa.201800111)

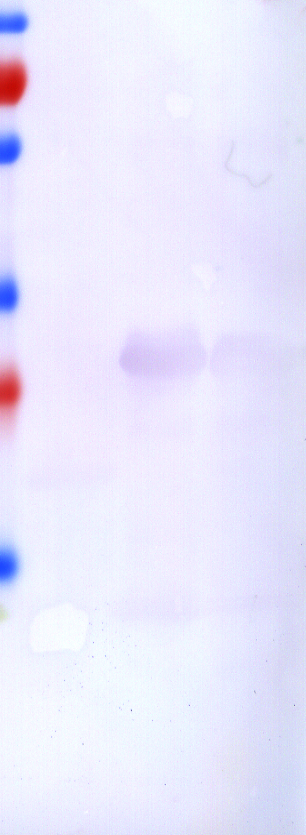

Supplement: Supplementary file 1 [file LSA-2018-00111_SdataF1.zip › LSA-2018-00111_SourceData_01.tif]

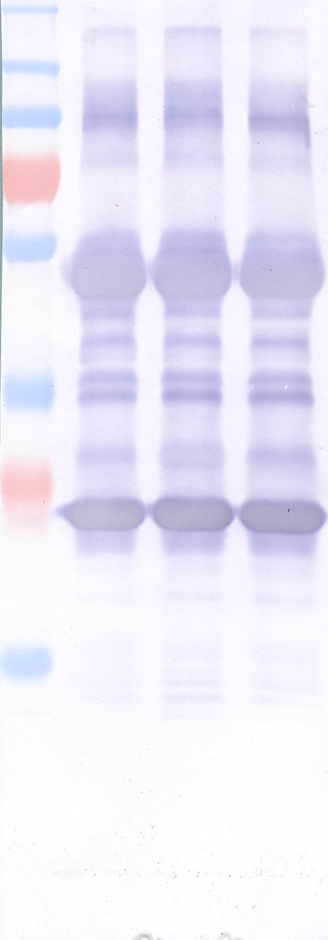

Supplement: Supplementary file 1 [file LSA-2018-00111_SdataF1.zip › LSA-2018-00111_SourceData_02.tif]

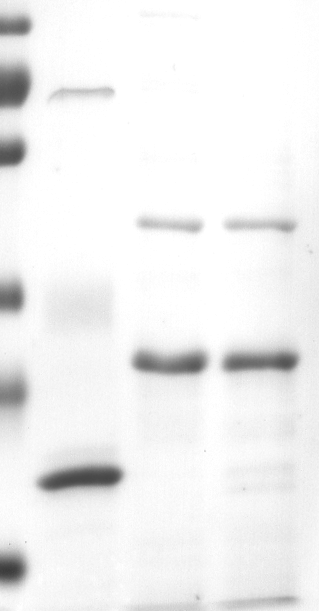

Supplement: Supplementary file 1 [file LSA-2018-00111_SdataF1.zip › LSA-2018-00111_SourceData_04.tif]

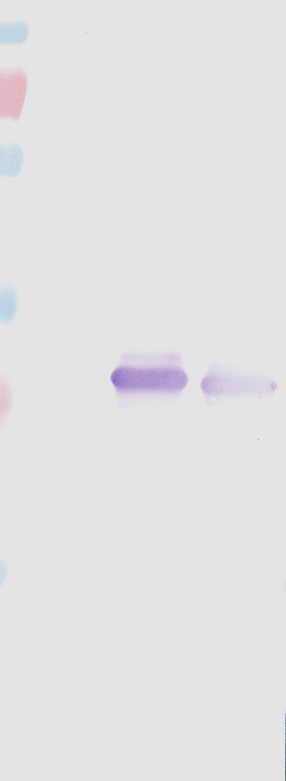

Supplement: Supplementary file 1 [file LSA-2018-00111_SdataF1.zip › LSA-2018-00111_SourceData_05.tif]
